# A Detailed Gene Expression Map of *Giardia* Encystation

**DOI:** 10.3390/genes12121932

**Published:** 2021-11-30

**Authors:** Laura Rojas-López, Sascha Krakovka, Elin Einarsson, Ulf Ribacke, Feifei Xu, Jon Jerlström-Hultqvist, Staffan G. Svärd

**Affiliations:** 1 Department of Cell and Molecular Biology, Uppsala University, SE-751 24 Uppsala, Sweden; laura.rojas@icm.uu.se (L.R.-L.); sascha.krakovka@icm.uu.se (S.K.); elineinarsson86@gmail.com (E.E.); feifei.xu@icm.uu.se (F.X.); jon.jersltromhultqvist@icm.uu.se (J.J.-H.); 2 Department of Microbiology, Tumor and Cell Biology (MTC), Karolinska Institutet, SE-171 65 Stockholm, Sweden; ulf.ribacke@ki.se; 3 SciLifeLab, Uppsala University, SE-751 24 Uppsala, Sweden

**Keywords:** diarrhea, RNAseq, small intestine, protozoa, differentiation

## Abstract

*Giardia intestinalis* is an intestinal protozoan parasite that causes diarrheal infections worldwide. A key process to sustain its chain of transmission is the formation of infectious cysts in the encystation process. We combined deep RNAseq of a broad range of encystation timepoints to produce a high-resolution gene expression map of *Giardia* encystation. This detailed transcriptomic map of encystation confirmed a gradual change of gene expression along the time course of encystation, showing the most significant gene expression changes during late encystation. Few genes are differentially expressed early in encystation, but the major cyst wall proteins CWP-1 and -2 are highly up-regulated already after 3.5 h encystation. Several transcription factors are sequentially up-regulated throughout the process, but many up-regulated genes at 7, 10, and 14 h post-induction of encystation have binding sites in the upstream regions for the Myb2 transcription factor, suggesting that Myb2 is a master regulator of encystation. We observed major changes in gene expression of several meiotic-related genes from 10.5 h of encystation to the cyst stage, and at 17.5 h encystation, there are changes in many different metabolic pathways and protein synthesis. Late encystation, 21 h to cysts, show extensive gene expression changes, most of all in *VSP* and *HCMP* genes, which are involved in antigenic variation, and genes involved in chromatin modifications. This high-resolution gene expression map of *Giardia* encystation will be an important tool in further studies of this important differentiation process.

## 1. Introduction

*Giardia intestinalis* is a significant cause of diarrheal disease worldwide in humans and other mammals [1]. This parasitic protozoan has been reported to cause more than 300 million cases of diarrhea in children less than five years old [2] and 28.2 million cases of foodborne infections, accounting for 26,270 disability-adjusted life years (DALYs) [3,4]. A key factor for the parasite to survive and establish infections in the next host is the ability to form infectious cysts: tetra-nucleate, dormant, and non-motile cells, which are highly resistant to environmental damage. *Giardia* alternates between the motile, binucleated, and disease-causing trophozoites that establish the infection in the upper small intestine and the cysts as the infective form [5].

The encystation process, differentiation of trophozoites to cysts, is triggered by environmental changes, such as cholesterol deprivation, bile content, and pH [6]; however, the specific molecular mechanisms regulating cell differentiation remain unclear. *Giardia* encystation can be divided into an early and late phase [6,7]. During the early encystation phase, the adhesive disc is disassembled [8], the flagella are internalized, the cells round up, and the cyst wall formation starts [6]. In addition, the formation of Encystation Specific Vesicles (ESVs) occurs, and ESVs transport the three major cyst wall proteins (CWPs 1–3) to the cell surface [9]. The CWPs make up most of the cyst wall, together with fibers of N-acetylgalactosamine, a sugar synthesized by enzymes, which are induced early in the encystation process [10]. In late encystation, division of the two nuclei occurs, DNA is replicated, and the maturation of the cyst wall is completed [6,11].

Previous studies using *in-vitro* and *in-vivo* transcriptomics and proteomics [12,13,14,15,16,17,18] have elucidated essential insights into the encystation process in *Giardia*. These investigations revealed changes in lipid and proteolytic metabolism and surface protein switching and suggested importance of chromatin remodeling factors during encystation. However, none of the previous studies has covered in detail the changes in gene expression going from trophozoites to cysts *in vitro* using several continuous timepoints of encystation, opening the question to what can be observed in a “mid-encystation” phase and how this is connected to the early and late phases. The earlier studies of changed RNA expression used methods with limited resolution, like microarrays [19], SAGE [12], or basic RNA sequencing [15], and only a few timepoints were included in the analyses. Here, we used our recently developed high-bile encystation protocol [15] and studied gene expression changes every 3.5 h of encystation using deep RNA sequencing (RNAseq). This new high-resolution gene expression data defines the early and late phases of *Giardia* encystation and show how they are connected, generating a high-resolution gene expression map that can be used in further studies of this important cellular differentiation process.

## 2. Materials and Methods

### 2.1. Cell Culture and Differentiation

Trophozoites of *G. intestinalis* isolate WB-C6 (ATCC catalog number 50803) were cultivated using TYI-S-33 medium supplemented with 10% adult bovine serum (Gibco, Thermo Fisher Scientific, cat. no 26170043, Uppsala, Sweden) and bovine bile (final concentration 0.125 mg/mL) at pH 6.8 as described previously [20]. Cells were cultivated in slanted polystyrene screw cap tubes (Nunc) to 80% confluency, medium was decanted, and changed for pre-warm encystation medium. Encystation was induced following the Uppsala encystation protocol as previously described [20].

### 2.2. RNA Extraction and RNA Sequencing

RNA was isolated from trophozoites and encysting cells at 3.5, 7, 10.5, 14, 17.5, 21, 24.5, 28, and 31.5 h post-induction of encystation (p.i.). At 31.5 h p.i., cysts were harvested and stored in ddH_2_O at 4 °C for three days prior RNA extraction. After three days storage, mature *in-vitro* cysts were excysted as described previously [15]. All samples were extracted using Trizol reagent as described by Franzén [21]. Three high-quality purified total RNA samples per timepoint were used for library preparation and RNA sequencing at the SNP/SEQ facility of the SciLifeLab National Genomics Infrastructure (Uppsala, Sweden) as described in [22].

### 2.3. Analysis of mRNA Sequencing Data

FASTQ files generated by the SciLifeLab were used for mapping in STAR v.2.5.4a with the latest version of the *G. intestinalis* WB genome [22] as reference. Raw files can be found at GEO [23] with accession number GSE141795 (https://www.ncbi.nlm.nih.gov/geo/query/acc.cgi?acc=GSE141795, (accessed on 11 December 2019)). For subsequent analysis STAR counts were fed into the edgeR [24] v3.26.8 workflow, and differential genes expression was established for all genes, with at least two million reads in three samples. Significance of expression changes were calculated with a quasi-likelihood F test using all three replicates of the T1 timepoint as control and comparing all three replicates of each other timepoint with it. For T2, only two replicates passed quality control. A cut-off of 5% FDR was used to choose the significantly differently expressed genes, which were used for subsequent analysis. Differentially expressed genes were used to detect functional clusters at different timepoints (3.5–7 h, 10.5–14 h, 17.5–21 h, 24.5–28 h, 31.5 h–Cyst, T2) by the Database for Annotation, Visualization, and Integrated Discovery (DAVID) annotation tool version 6.8 [25,26].

### 2.4. DNA Motif Analyses

The upstream region (−200 bp) of differentially expressed genes at 3.5, 7, 10.5, and 14 h p.i. were scanned for enriched motifs using XSTREME [27]. The upstream region of 149 genes not showing differential regulation during encystation was used as control sequences (Table S3). CentriMO was used to display the placement of CWACAG motifs in the upstream regions of differentially upregulated genes [28]. FIMO was used to search for occurrences of the Myb2 binding motif CWACAG in the upstream regions of differentially upregulated WB genes at 3.5, 7, 10.5, and 14 h p.i. and in the corresponding GiardiaDB [29] orthologs in *G. intestinalis* assemblage E isolate P15, assemblage B isolate GS, and *G. muris* (Table S3) [30]. The orthologous upstream regions for *G. intestinalis* assemblage E isolate P15, assemblage B isolate GS, and *G. muris* at 7 and 10.5 h were scanned by XSTREME as above to detect Myb2-like binding motifs.

### 2.5. Analyses of Double-Stranded DNA Breaks

Cells of trophozoites and encysting cells at 3.5, 7, 10.5, 14, 17.5, 21, and 24.5 h p.i. were harvested by keeping them on ice for 15 to 20 min. The cells were detached by tapping followed by centrifugation (500× *g*; 10 min; 4 °C). The supernatant was removed, and the cells were prepared either for microscopy or Western Blot. A stock solution of 1 mg/mL aphidicolin from *Nigrospora sphaerica* (Sigma, Stockholm, Sweden) was prepared, aliquoted, and stored at −20 °C. Trophozoites were incubated with 5 μg/mL aphidicolin to be used as control for double-stranded breaks [31].

Samples for Western Blot were prepared as described by [32]. Protein samples were separated on SDS-PAGE gels (Any kD, Mini-PROTEAN TGX, Bio-Rad, Hercules, CA, USA) and transferred to PVDF membranes by electroblotting using standard techniques. Double-stranded breaks were detected using an antibody against phosphorylated Ser139 in human histone H2AX (AR-0149, LP BIO) [31]. Membranes were blocked with PBS containing 0.1% Tween-20 and 3% skim milk for 1 h at room temperature. After washing with Tris-buffered saline (TBS), membranes were incubated with the H2AX primary antibody in a dilution of 1:1000 at 4 °C overnight. Bound antibodies were detected with horseradish peroxidase conjugated secondary antibody (anti-rabbit HRP, Thermo Scientific, Rockford, IL, USA) diluted 1:10,000 in 3% milk. The blots were developed by using Clarity Western ECL Substrate (Bio-Rad), and images were recorded on a Chemi-Doc MP (Bio-Rad, Hercules, CA, USA).

Samples for immunofluorescence assays were washed three times with cold PBS and put as monolayers on poly-L-lysine-coated multi-well slides (Thermo Fisher Scientific). The cells were fixed and prepared following the method described in [15]. To detect double-stranded DNA breaks the samples were incubated with the H2AX antibody as primary antibody and diluted 1:250 in 2% BSA/0.1% Triton-X100/PBS for 90 min. After washing the wells six times with PBS, the secondary antibody anti-rabbit Alexa Fluor 594 (1:800) in 2% BSA/0.1% Triton-X100/PBS was added and left to incubate for 60 min. Additionally, the monoclonal mouse anti-CWP1 antibody conjugated with Alexa Fluor 488 (Waterborne Inc., New Orleans, LA, USA) was used at dilution 1:100. Thereafter, the washing procedure was repeated and the slides were mounted in Vectashield containing DAPI (Cat. no. H1-100, Vector Laboratories, Burlingame, CA, USA). The slides were viewed in a Nikon Ti Eclipse fluorescence microscope, and images were processed using the NIS-Elements imaging software. For each condition, at least 100 cells were studied, and the number of cells with a positive signal for H2AX were estimated, and the percentage of positive cells against total cells was calculated. 

## 3. Results

### 3.1. Overall Gene Expression Changes during Encystation

**Figure 1:**
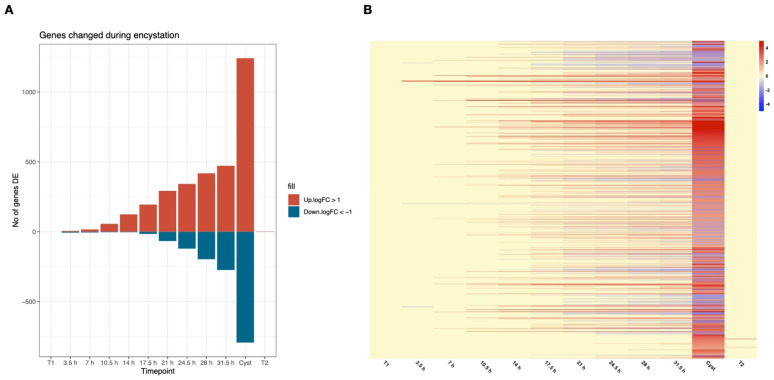
Overall gene expression changes during *Giardia* encystation: (**A**) Number of significantly differentially expressed genes (DEGs) compared to starting trophozoites (T1) with a log2-fold change of more than one, which equals a fold change of 2 or bigger separated by timepoints; (**B**) heat-map of log2–fold changes of the top 1000 most changed DEGs in cysts including all timepoints. Changes are capped at -5 and 5, respectively, to improve visualization. Up-regulation is shown in red, while down-regulation is shown in blue.

In order to get more detailed information about gene expression changes during *Giardia* encystation, we used deep RNAseq combined with the newly annotated and more complete *G. intestinalis* WB genome [22] to generate a comprehensive, high-resolution map of *Giardia*’s gene expression profile during encystation. Several different *in-vitro* encystation protocols exist, but we used our recently developed high-bile protocol called the Uppsala encystation, which is more efficient in generating mature cysts [15]. Expression was analyzed every 3.5 h throughout the process, starting with trophozoites (T1), 3.5, 7, 10.5, 14, 17.5, 21, 24.5, 28, and 31.5 h post-induction of encystation (p.i.) and mature cysts after three days water treatment. We also generated data from trophozoites established after excystation of cysts (T2). The transcriptome data is summarized in Figure 1 and Table 1 and Table S1, and it shows an increase in differentially expressed genes (DEGs) that are up- and down-regulated along the time-course of encystation. There is a major difference towards the end of the encystation process (Figure 1A); after 3.5 h encystation, there are 20 DEGs (six up- and 14 down-regulated genes), whereas there are 3409 DEGs in the cyst stage (1786 up- and 1623 down-regulated genes). The top 1000 most DEGs in cysts during the different timepoints are shown in a non-clustered heatmap (Figure 1B), and this shows the gradual induction of genes highly expressed in cysts, in line with earlier data [15]. The increased depth of sequencing and tighter timepoint intervals resulted in a much higher resolution in the encystation transcriptional map compared to earlier studies (Figure 1 and Table 1).

An analysis of up- and down-regulated genes at the different timepoints (Table S1) showed big similarities between the timepoints, and we decided to cluster the data in six groups in further analyses (3.5–7 h, 10.5–14 h, 17.5–21 h, 24.5–28 h, 31.5h–Cyst, and T2) to simplify the presentation of data. The DAVID algorithm was first used to investigate function-related gene groups via GO term analyses in the different clustered timepoints. The cluster analysis was performed on DEGs, and only clusters with enrichment scores of ≥1.3 were included (Table S2). The DAVID analysis data are summarized in Table 2 and highlight certain cellular processes with DEGs at specific encystation stages. Note that during timepoints 3.5–7 h and 10.5–14 h, no significant changes were found in the down-regulated genes. In the early timepoints 3.5 and 7 h, EGF domain proteins are enriched in the up-regulated DEGs, and they are all high cysteine-rich membrane proteins (HCMPs, Table S2). It is clear from this analysis that changes in lipid metabolism are associated with the encystation process, in line with earlier data (Table 2 [33]). In the cysts and excysted trophozoites, *Variable Surface Protein* (*VSP*) genes that are involved in antigenic variation are the main DEGs. The down-regulated genes show no significant enrichment of genes from a certain class early in encystation, but from 17.5 h, there is an enrichment of genes involved in nucleotide binding and protein synthesis (Table 2). 

**Figure 2:**
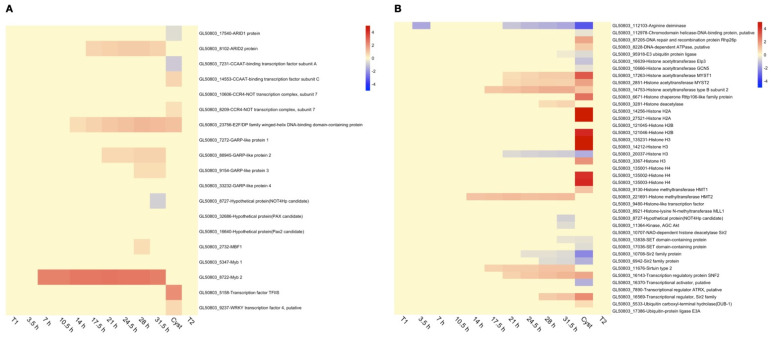
Heat-map showing expression changes through-out encystation of (**A**) transcription factors and (**B**) chromatin modifiers. All changes are in log2 space and capped at −5 and 5, respectively, to improve visualization. Up-regulation is shown in red, while down-regulation is shown in blue. For each gene, the unique Geneid (starting in GL50803_) and the annotated function is given.

In the earlier studies of *Giardia* encystation, RNA expression profiles known and putative transcription factors were investigated as well as chromatin modifiers, such as histone deacetylases (HDACs), histone methyltransferases (HMTs), and histone acetyltransferases (HATs) together with putative chromatin remodeling complex members [15]. We decided to follow this up by studying the expression of known giardial transcription factors (Figure 2A) and chromatin modifiers (Figure 2B) to have a more detailed picture of these transcripts’ temporality during the encystation process. The non-clustered heatmap of the transcription factors shows an early activation of the previously identified encystation-associated transcription factor Myb 1-like protein or Myb2 as it has been called (GL50803_8722, Figure 2A), which confirms its key role in the early phases of encystation [13,15,34]. Several other transcription factors (GARP, ARID, Pax, WRKY, and E2F family) have been described as to be involved in the up-regulation of genes important in early encystation [34], but none of them were observed to be up-regulated on the RNA level during the first 7 h of encystation (Figure 2A). However, there is instead a stepwise upregulation of these factors: EF2/DP (GL50803_23576) at 10.5 h p.i., ARID2 (GL50803_8102) at 14 h p.i., GARP-like 2 (GL50803_88945) at 21 h, and GARP-like 3 protein (GL50803_9154) at 28 h p.i. (Figure 2A). None of the chromatin-modifying proteins were differentially expressed early in encystation (first 10 h, Figure 2B), but from 14 h p.i. (starting with histone methyltransferase HTM2, GL50803_221691) towards mature cysts, there are more and more signs of changes in chromatin modifying factors and histones in the mature cysts (Figure 2B). Thus, our high-resolution data suggest an initial transcription factor cascade type of gene regulation starting early in encystation with Myb2 having a key role, and this is followed by changes in chromatin modifying factors and histones in mid to late encystation (Figure 2B). This results in large gradual changes in RNA expression of protein-encoding genes going from trophozoites to cysts, in the end resulting in that 69% (3409/4963) of the protein-encoding genes in *Giardia* are DEGs [22]. Interestingly, when the cysts are excysted and cells are regrown to confluent trophozoites (T1 trophozoites compared to T2 trophozoites), only three DEGs are found, and all are VSPs (two up- and one down-regulated VSP, Table 1 and Table S1). 

### 3.2. Gene Expression Changes in Early Stages of Encystation (3.5 and 7 h)

After 3.5 h of growth in encystation medium, only six genes were observed to be significantly upregulated (Table 1 and Table S1). Among this small selection of early up-regulated genes, the most robust up-regulation was observed in the two major *cyst-wall proteins-1 and -2 (CWP-1 and -2)* [35,36,37]. A hypothetical protein with similarities to cyclin (GL50803_15532) was observed as part of the early up-regulated genes. The expression profile shows a cyclic expression profile during encystation with increased expression at 3.5 h and 21 h to cysts, which is unique among the giardial cyclins (Table S1 and Figure S1). Previously, the role of the cyclin-dependent kinase Cdk2 (GL50803_16802) during encystation has been investigated and has been proven to affect phosphorylation of Myb2 and the expression of CWP-1 and -2 and cyst formation [38]. Thus, it is possible that this cyclin-like protein interacts with cdk2 and regulates encystation via phosphorylation of Myb2. 

Three genes encoding *HCMPs* (GL50803_9276, 11,309, and 25,816) show up-regulation early in encystation (Figure S2, Table S1), and all three *HCMP* genes are among the most up-regulated genes when Cdk2 is overexpressed in trophozoites [38]. The early up-regulation of these three HCMPs on the RNA level has also been seen in earlier expression studies of encystation [13,15], but none of the other HCMPs show this expression profile (Figure S2).

When it comes to down-regulation, 14 genes showed decreased expression at the first 3.5 h p.i. of encystation (Table 1 and Table S1). Among these genes, the seven-transmembrane protein, the major facilitator superfamily transporter, the glycosaminoglycan polysaccharide lyase, the carbamate kinase, and the arginine deiminase can be found. Additionally, the down-regulation of two *HCMP*s (GL50803_41942 and GL50803_39904, Figure S2) and seven hypothetical proteins was observed (Table S1). Among the hypothetical proteins, we have early down-regulation of a protein with a cyclin-like domain (17241) and two putative nuclear proteins (GL50803_5908 and GL50803_31144). The gene encoding hypothetical protein GL50803_5908 is part of a gene family in the WB genome with 17 members, but only this gene in the family is up-regulated early in encystation.

At 7 h p.i. of encystation, there are 21 genes significantly up- and 12 down-regulated (Table 1 and Table S1). Only CWP-1 and -2 are overlapping in the up-regulated gene set from the 3.5 h timepoint, but the Myb1 transcription factor and CWP-3 are highly up-regulated at this timepoint. The *Giardia* cyst-wall is composed of 37% protein (mainly CWP1 to 3) and the sugar N-acetylgalactosamine, which is synthesized by an encystation-inducible pathway [10]. Already, at this timepoint, there is an up-regulation of enzymes involved in carbohydrate metabolism (the transglycosidase CEGP1 (GL50803_17210), glucose 6-phosphate N-acetyltransferase (GL50803_14259), and phosphoglucosamine mutase (GL50803_16069)). 

Biotin protein ligase, BPL, is responsible for the post-translational attachment of biotin to a specific lysine residue present in the active site of biotin-dependent enzymes [39]. The giardial BPL enzyme is robustly up-regulated throughout the encystation process (Table S1). Acetyl-CoA carboxylase (ACC) and pyruvate carboxylase (PC) are well-characterized enzymes that are regulated by biotinylation. ACC is a critical enzyme for the carboxylation of acetyl-CoA to malonyl-CoA in fatty acid biosynthesis, and biotin-activated PC is involved in the conversion of pyruvate to oxaloacetate [39]. Interestingly, these two enzymes are fused into a large 1338 amino acid protein in *Giardia* (GL50803_113021) with a biotin carboxyl carrier domain [40], and the gene is up-regulated at 14 h of encystation (Table S1). These data suggest a putative role of biotin (vitamin B_7_) in the regulation of encystation. 

Earlier studies of encystation showed a role of S-protein palmitoylation [41], and here, we see an early up-regulation of a DHHC palmitoyl transferase (GL50803_2116). This protein is specific for encysting cells, as shown in a recent proteomic analysis of encystation [16]. Cyst wall proteins are transported in encystation-specific vesicles (ESVs) to the cell surface, and several proteins putatively involved in these processes are induced at 7 h (Hypothetical protein GL50803_33672, Synaptic glycoprotein SC2 (GL50803_88581). The 12 down-regulated genes at 7 h show a large overlap with the genes downregulated at 3.5 h, except for the nitroreductase domain containing protein GL50803_6175 and the Flap endonuclease XPG family protein (GL50803_14208).

**Figure 3:**
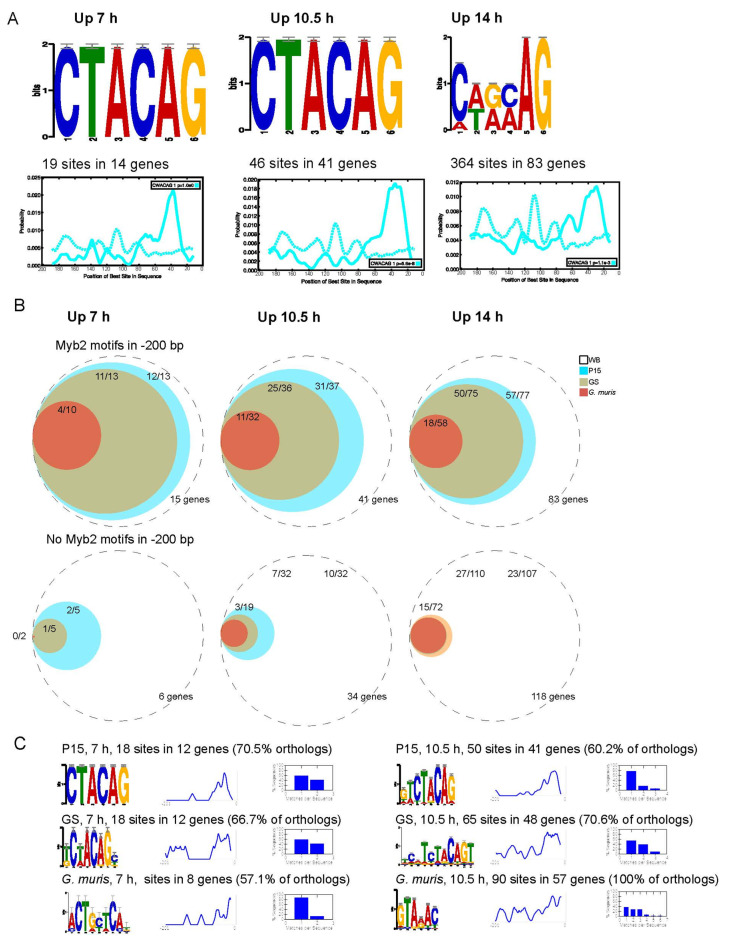
Analysis of Myb2 like motifs. (**A**) Enriched Myb2-like motifs (top panel) and their position in the upstream region (lower panel) of differentially upregulated genes at 7, 10.5, and 14 h p.i. (**B**) Upregulated WB genes with canonical Myb2-sites (CWACAG) are more likely to also have a Myb2-site in the ortholog of assemblage E (P15) (teal), B (GS) (gold), and in *G. muris* (red). (**C**) De-novo motif finding in the orthologs of upregulated WB genes in assemblage E (P15) (top), B (GS) (middle), and in *G. muris* (bottom) at 7 and 10.5 h p.i.

Previous studies of *Giardia* encystation identified a small consensus set of 13 genes that are up-regulated during early encystation [13,15]. Most of the upstream region of these genes contains the hexamer CWACAG, which has been experimentally verified to be a Myb2-binding sequence [13,34]. A similar motif has also been reported upstream of some orthologs of encystation-related genes in *G. muris* [42]. We decided to investigate the presence of Myb2-like motifs upstream of up-regulated genes in our data set. At 7 h p.i., we found the canonical Myb2-motif in the upstream region (−200 bp from start codon) in 14 out of 21 up-regulated genes. The size of the Myb2-regulon increased to 41 genes out of 75 up-regulated genes at 10.5 h. At 14 h post-induction, the set of up-regulated genes were still enriched in a hexanucleotide sequence with some resemblance to the Myb2-interacting motif (Figure 3A). The CWACAG motif was found upstream of 83 genes up-regulated at this time-point. Most genes have a single instance of the motif, but additional copies are present for some genes. At 7.5 h, we found that the Myb2-motifs were positioned in a 15 bp region on average 41.5 bp from the start codon. The preferential position of the Myb2-motif did not shift greatly in genes up-regulated at later time-points, but the region of occupancy increased in size somewhat (Figure 3A). To investigate the evolutionary stability of the Myb2-regulon, we checked the upstream region to orthologs of the regulated genes in other *Giardia* assemblages (B and E) and *Giardia muris*. We found that many orthologs to encystation-regulated genes in *Giardia* assemblage B (GS isolate) and E (P15 isolate) maintained a putative Myb2-site and that the proportion of conserved sites decreased by genetic distance from *G. intestinalis* assemblage A (Table S3). Most of the up-regulated genes lacking a Myb2-site in WB also lack such binding sites in the upstream regions of orthologs in the other assemblages (Figure 3B). There are however some notable exceptions where canonical Myb2-motifs are detectable in the P15 and GS isolates but are absent in the WB isolate (GL50803_40376—High cysteine non-variant cyst protein, GL50803_16217—UDP-N-acetylglucosamine pyrophosphorylase, GL50803_9352—U5 small nuclear ribonucleoprotein 200 kDa helicase, putative). De-novo motif searches suggests that degenerate variants of the CWACAG motifs, if positioned correctly, might be able to induce genes. Moreover, the de-novo motif finding in assemblage E and B orthologs of encystation-induced genes recovers enrichment of motifs reminiscent, but with slight variation, of the Myb2-binding site (Figure 3C). However, *G. muris* did not show as prominent motif and position conservation in its set of orthologs. To conclude this analysis, the number of genes in the Myb2-regulon in *G. intestinalis* appear to be larger than previously appreciated and is conserved in other *G. intestinalis* assemblages.

### 3.3. Gene Expression Changes in Early to Mid Stages of Encystation (10.5 and 14 h)

After 10.5 h of encystation, there are 79 up-regulated genes and only nine down-regulated genes that mostly overlap with the 3.5 h timepoint (Table 1 and Table S1). At 14 h of encystation, there are 200 up-regulated genes and 23 down-regulated genes (Table 1 and Table S1). The DEGs suggest that here are some major changes in certain cellular process, and most genes needed for cyst formation peak at this stage of encystation. First, all the major enzymes involved in synthesis of the N-acetylgalactosamine in the *Giardia* cyst-wall are highly induced at this stage of encystation (glucose 6-phosphate N-acetyltransferase (GL50803_14259), glucosamine-6-phosphate deaminase (GL50803_8245), phosphoglucosamine mutase (GL50803_16069), UDP-glucose 4-epimerase (GL50803_7982), and UDP-N-acetylglucosamine pyrophosphorylase (GL50803_16217)), at the same time as the cyst wall proteins CWP-1 to -3, and High cysteine non-variant cyst protein HCNP (GL50803_40376, Figure S2) establish a high level of expression that is kept throughout the encystation process (Table S1). 

Second, there are large changes in the lipid metabolism (Table 2), probably reflecting that the levels of ESV formation peak around 14 h in this encystation medium [15]. The genes encoding endoglycosylceramidase (GL50803_12066) and ceramide glucosyltransferase (GL50803_11642) are highly up-regulated, and glucosyl- and galactosylceramide are important in ESV formation and cyst viability [43]. Another DHHC palmitoyltransferase (GL50803_96562) is up-regulated, and there are also changes in gene expression of many enzymes involved in long chain fatty acids (Figure S3). 

**Figure 4:**
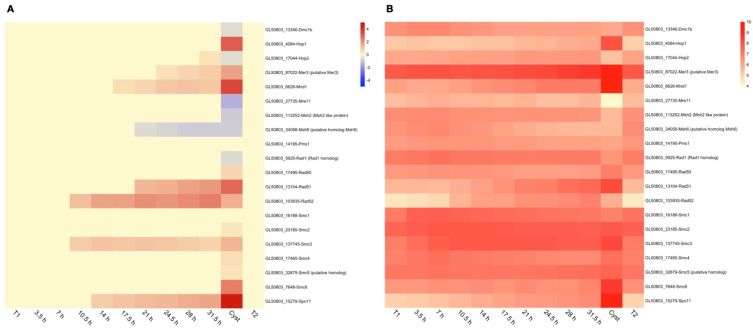
Heat-maps showing expression through-out encystation of meiosis-related genes. (**A**) Expression changes compared to trophozoites (T1) and (**B**) relative expression levels of meiosis-related genes using count per million (CPM) values. Expression changes are in log2 space and capped at -5 and 5, respectively, to improve visualization. Up-regulation is shown in red, while down-regulation is shown in blue. CPM values are in log2 space and capped at 10 CPM to improve visualization. For each gene, the unique Geneid (starting in GL50803_) and the annotated function is given.

Third, genes involved in DNA recombination/meiotic processes are induced at 10.5 and 14 h of encystation (Figure 4). This is supported by an increase of double-stranded DNA breaks and the up-regulation of the H2AX protein around the same time (Figure S4). Earlier studies have shown that the *Giardia* genome contain genes that are usually involved in meiosis [44], but neither meiosis nor sexual stages have been identified. At least three of the genes (*Spo11*, *Hop1*, and *Rad51*) are only expressed and localize to the nuclei during encystation [45]. The two nuclei in *Giardia* fuse during encystation, and there is DNA exchange via homologous recombination [45,46]. Here, we can see a temporal expression of the meiotic related genes (Figure 4). A putative cohesin (GL50803_137745), which is usually involved in the alignment of sister chromatids in the first step of meiotic recombination, is up-regulated at 10.5 h together with Rad52, a DNA repair protein that interacts with Rad51 (Figure 4). At 14h of encystation, Spo11 is induced, and the enzyme producing double-stranded breaks in one of the sister chromatids during meiosis. Mnd1 and Rad51 are up-regulated at 17.5 and 21 h, respectively (Figure 4). Several of the meiotic-related genes change their expression in the cyst stage, including *Hop1*, *Mre11*, and *Smc6*, suggesting that the process continues until the end of the differentiation process. This high-resolution gene expression map can be the basis for further studies of this meiotic-like process. 

### 3.4. Gene Expression Changes in Mid Stages of Encystation (17.5 and 21 h)

In mid encystation, there are large changes in gene expression: at 17.5 h, 382 up- and 130 down-regulated genes and at 21 h, 598 up- and 414 down-regulated genes (Figure 1A, Table 1). At the same time as the earlier noted gene expression changes associated with cyst wall synthesis occur, there are also new changes in many different metabolic pathways and protein synthesis. Thus, mid encystation seems to be an important transition in the *Giardia* life cycle. 

Several changes in energy metabolism can be detected. Fructose-2,6-bisphosphate biosynthesis is upregulated (Histidine phosphatase superfamily proteins GL50803_135885 and 135886), as is the oxidative branch of the pentose phosphate pathway (6-phosphogluconate dehydrogenase, decarboxylating GL50803_14759, and 6-phosphogluconolactonase/Glucose-6-phosphate 1-dehydrogenase GL50803_8682). Aspartate aminotransferase (GL50803_91056), which is involved in anaerobic energy metabolism, is up-regulated. At the same time, there is a down-regulation of L-citrulline degradation (Carbamate kinase GL50803_16453) and L-serine degradation (L-serine dehydratase GL50803_24662). The glycolytic enzyme Glyceraldehyde 3-phosphate dehydrogenase (GAP-2, GL50803_17043) is highly up-regulated at this stage (Table S1 and Figure S3). In the *Giardia* genome, two genes are encoding GAPDH: one is constitutively expressed (*GAP-1*, GL50803_6687), and one is up-regulated in mid encystation (*GAP-2*) [47]. Only GAP-1 has been shown to have GAPDH activity, making it the main enzyme used in glycolysis, whereas GAP-2 lacks GAPDH activity [47]. The GAP-2 protein is highly divergent to GAP-1 (51% amino acid identity and several insertions), and the sequence most likely diverged after the separation of the *Giardia* linage from the other diplomonads [48]. 

Metronidazole is the main drug for treatment of giardiasis, and it is a pro-drug that is activated by oxidoreductases in *Giardia* [49]. The main enzyme suggested to be involved in the activation of metronidazole in *Giardia* is the nitroreductase NR1 (Nitroreductase Fd-NR2, GL50803_22677). At 21 h of encystation, this gene is down-regulated, possibly explaining the earlier observed reduced sensitivity of late encysting *Giardia* cells and cysts to metronidazole compared to trophozoites [50]. Co-factors like NADH and NADPH and oxidative stress responses have also been suggested to be important in metronidazole resistance, and there are also gene expression changes in the NAD salvage pathway (up-regulation of nicotinamide-nucleotide adenylyltransferase, GL50803_92618, and NH_3_-dependent NAD+ synthetase, GL50803_31530) and glutathione biosynthesis (up-regulation of glutamate-cysteine ligase, GL50803_16001, and glutathione synthetase, GL50803_15429), which can affect *Giardia*’s sensitivity to metronidazole.

*Giardia* parasites need to salvage nucleosides from the host, and there is an additional DNA replication step at the end of encystation that is needed to obtain mature 16N cysts [11]. At 17.5 and 21 h of encystation, most nucleoside salvage pathways are down-regulated, and this continues until the end of encystation: guanine and guanosine salvage (guanine phosphoribosyl transferase, GL50803_6436, and nucleoside ribosyl transferase/purine nucleoside phosphorylase, GL50803_91348), purine deoxyribonucleosides degradation (adenylate kinase/UMP-CMP kinase, GL50803_90402), pyrimidine ribonucleosides salvage (uridine kinase, GL50803_8217 and CTP synthase, GL50803_17587), and methyl-5’-thioadenosine_degradation (5’-methylthioadenosine nucleosidase/S-adenosylhomocysteine nucleosidase, GL50803_20195 and 5’-methylthioadenosine nucleosidase/S-adenosylhomocysteine nucleosidase, GL50803_4059). On the contrary, the cell-cycle regulated genes that are involved in DNA replication thymidylate kinase (GL50803_15380), replication factor C, subunits 5 (GL50803_16127), DNA replication licensing factor MCM2 (GL50803_15344), and MCM3 (GL50803_16214) are up-regulated between 21 and 28 h of encystation, suggesting that a large part of the cells in the encysting population replicate the DNA at the end of encystation.

**Figure 5:**
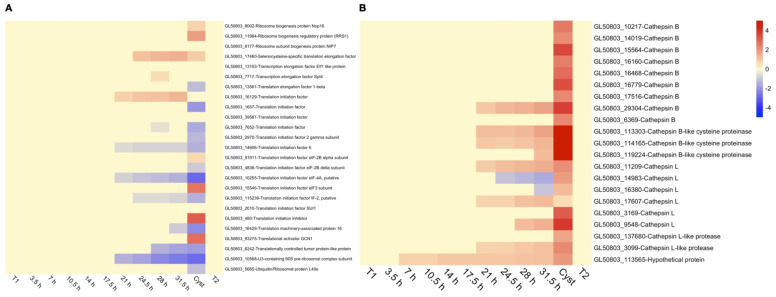
Heat-maps showing expression changes through-out encystation of (**A**) translation initiation factors and (**B**) cathepsins. Expression changes are in log2 space and capped at -5 and 5, respectively, to improve visualization. Up-regulation is shown in red, while down-regulation is shown in blue. For each gene, the unique Geneid (starting in GL50803_) and the annotated function is given.

Protein synthesis is down-regulated at 17.5 and 21 h of encystation. This is reflected in the down-regulation of 9 tRNA synthetases (arginyl-tRNA synthetase, GL50803_10521, glutamine-tRNA synthetase, GL50803_9348, valine-tRNA ligase, GL50803_35428, alanyl-tRNA synthetase, GL50803_96460, seryl-tRNA synthetase, GL50803_101501, phenylalanyl-tRNA synthetase beta chain GL50803_101501, phenylalanyl-tRNA synthetase alpha chain, GL50803_12162, lysyl-tRNA synthetase, GL50803_16766, histidyl-tRNA synthetase, GL50803_22808, and methionyl-tRNA synthetase, GL50803_22204). The effect is also seen on translation initiation factors (Figure 5A) and ribosomal proteins (Figure S5). At the same time as protein synthesis is down-regulated, proteolytic activities are increased (Figure 5B). Six cathepsins are up-regulated from 21 h of encystation until the end of encystation, and 11 are up-regulated in cysts (Figure 5B). A recent study of *G. intestinalis* encystation using proteomics confirm that there are large changes in the proteome in the end of encystation [16].

### 3.5. Gene Expression Changes Late in Encystation (24.5 to 31.5 h)

There are extensive changes in gene expression in the end of encystation (Figure 1 and Table 1), and among the most significantly changed genes, we find genes involved in lipid metabolism and antigenic variation (variable surface proteins, VSPs and HCMPs). 

The cyst wall is built on the surface of the trophozoites plasma membrane, and there is an additional membrane layer synthesized that eventually will be the surface of the excyzoite, the parasite-stage that emerges from the excysting cyst [11,51]. Enzymes involved in CDP-diacylglycerol biosynthesis (Lysophosphatidic acid acyltransferase/Glycerol-3-phosphate 1-*O*-acyltransferase (GL50803_14403)) and phospholipid remodeling (down-regulation of phospholipase B GL50803_9354 and GL50803_17277 and glycerophosphocholine phosphodiesterase (GL50803_6492)) are differentially expressed at this stage. Phosphorylated forms of the lipid phosphatidylinositol (PIPs) are involved in membrane dynamics like clathrin-mediated endocytosis (CME). In *Giardia,* it has been shown that PIs and PIP-binding proteins are important for endocytic uptake via plasma membrane invaginations that are in contact with the specialized giardial endocytic organelles called peripheral vacuoles (PVs) [52]. There are major changes in 3-phosphoinositide biosynthesis (up-regulation of CDP-diacylglycerol-inositol 3-phosphatidyltransferase (GL50803_9829), phosphatidylinositol-4-phosphate 5-kinase (GL50803_11897), and (GL50803_13606)) and degradation (up-regulation of Phosphatidylinositol-3,4,5-trisphosphate 3-phosphatase (GL50803_16728), Type II inositol-1,4,5-trisphosphate 5-phosphatase (GL50803_14787), and Myotubularin-like protein (GL50803_8210)). Earlier studies showed that the PI-3 kinase GL50803_17406 is up-regulated during encystation [53], but in this study, it was expressed at constant levels (Table S1). 

**Figure 6:**
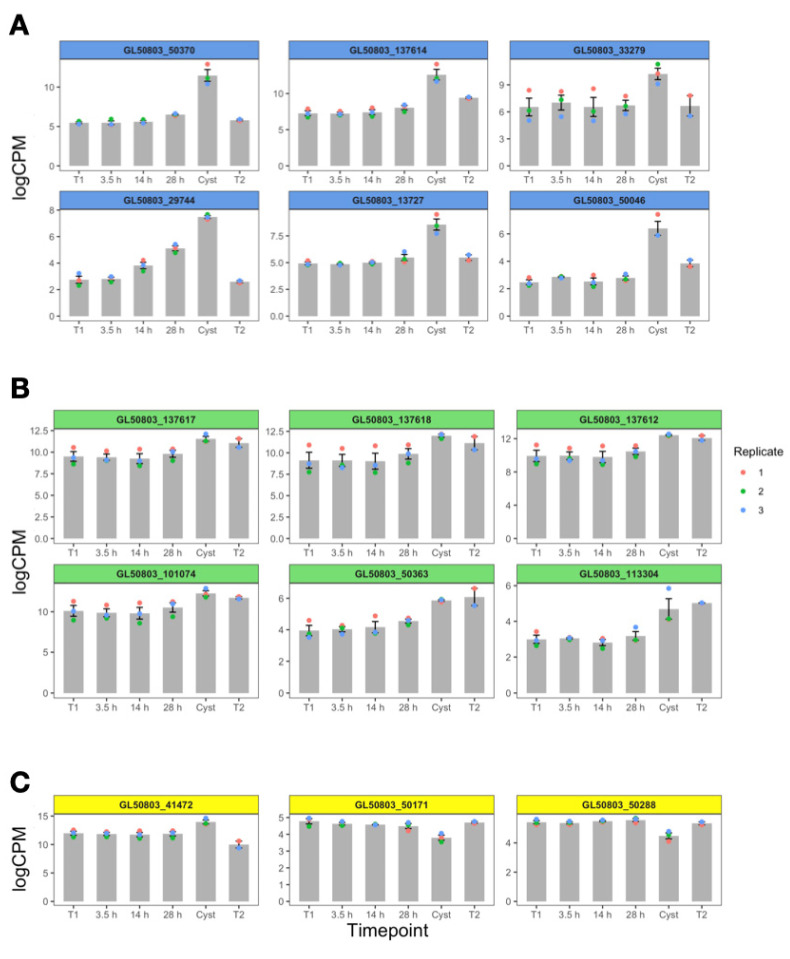
VSP expression during selected timepoints of encystation as CPM values. Red dot, replicate 1; green dot, replicate 2; blue dot, replicate 3. CPM values are in log2 space. The genes were assigned into three different groups with similar behavior during encystation: (**A**) Genes with a blue header were found to be most upregulated in the cyst when compared to T1 and reversed to T1 levels in T2; (**B**) Genes with a green header were up-regulated in both cyst and T2 when compared to T1; (**C**) Genes with a yellow header where down-regulated in T2 and/or cyst when compared to T1.

*Giardia* has a large repertoire of cysteine-rich surface proteins, including VSPs, pVSPs, and HCMPs [22]. The *HCMP* genes encode proteins that localize to the PM but also to internal membranes [54], and they show many different expression patterns throughout encystation (Figure S2). The VSPs have been shown to be surface localized and to be involved in antigenic variation. The group pVSP was created since no clear secretion signals were detected in the *pVSP* genes [42]. Here, we can see that certain VSPs are induced during encystation, when the expression of the starting VSPs are reduced. The 10 most highly induced *VSP* genes during encystation can be seen in Figure S6, and all have been seen to be induced during encystation in our earlier RNAseq study [15]. Many *VSP*s are up-regulated in cysts in the three biological replicates (Table S1 and Figure 6), which could be a preparation for excystation, making newly excysted cells antigenically different compared to the starting trophozoite population. However, a few VSPs are also down-regulated in cysts (Figure 6). Thus, there seems to be a set of *VSP* genes that are encystation-specific, and there are other *VSP* genes that show different types of expression profiles during encystation. This shows a connection between differentiation and antigenic variation in *Giardia*.

### 3.6. Gene Expression Changes in Cysts and Excysted Cells (C and T2)

The largest gene expression differences from the starting trophozoites are, as expected, seen in the cysts with 3409 DEGs (1786 up-regulated and 1623 down-regulated genes, Figure 1A and Table 1). Plotting expression over the whole encystation process for the top 1000 DEGs in cysts (Figure 1B) show a gradual change of gene expression but large differences between 31.5h and cysts. *Giardia* cysts are not metabolically inactive, but they have a much lower energy consumption than trophozoites and encysting cells [50]. This can be seen in the gene expression data, where most glycolytic enzymes are down-regulated in the cysts, as are genes in the arginine deaminase pathway (Table S1). Thus, the two main pathways for energy generation are down-regulated. Down-regulation can also be seen in genes involved in anaerobic energy metabolism (pyruvate kinase (GL50803_17143 and 3206), alanine aminotransferase (GL50803_12150), malate dehydrogenase (GL50803_3331 and 14285), and phosphoenolpyruvate carboxykinase (GL50803_10623). The malate-aspartate shuttle pathway (aspartate aminotransferase, GL50803_91056 and malate dehydrogenase, GL50803_3331) and pyruvate fermentation to ethanol are also down-regulated in cysts (Table S1). However, at the same time, one of the two alanine aminotransferase genes is highly up-regulated (GL50803_16363, Table S1). *Giardia* trophozoites can take up glutamate from the medium and glutamate dehydrogenase, and alanine aminotransferase activities cooperate in the conversion of pyruvate to alanine and at the same time consume NAD(P)H [55]. During the whole encystation process and in cysts, NADP-specific glutamate dehydrogenase (GL50803_21942) is highly up-regulated. The synthesis of alanine, and consumption of NAD(P)H, is sensitive to the surrounding oxygen levels, as it mainly occurs at low oxygen levels [56]. This is a condition seen in the lower part of the intestine, where *Giardia* cysts are formed and matured. The NAD+-producing enzyme nicotinamide-nucleotide adenylyltransferase (GL50803_92618) is also highly up-regulated in cysts, suggesting that there is a shift in the redox balance in cysts compared to earlier in the life cycle. The low metabolic activity in cysts is also reflected in the down-regulation of genes involved in in protein synthesis (Figure S3), nucleoside metabolism, and cytoskeleton proteins, including the adhesive disc (Table S1). 

The largest group of up-regulated genes in cysts are hypothetical proteins (Table S1), and the two most up-regulated genes are also hypothetical proteins (GL50803_11050 and 5206). Both genes show up-regulation after 14 h of encystation, and the level of up-regulation increases throughout encystation. The protein encoded by ORF11050 is 122aa, has two predicted membrane-spanning regions, has a pI of 10.27, and it is not found in the *G. muris* genome. The protein encoded by ORF5206 is 255aa, has six membrane-spanning regions and is part of a small gene family with six members, all small proteins with six membrane-spanning proteins being up-regulated at different stages of encystation. Further studies will show what roles these proteins play during encystation.

All histone variants are highly up-regulated on the RNA level in cysts (histone H2A (GL50803_14256 and 27521), histone H2B (GL50803_121046), histone H3 (GL50803_14212 and 135231), and histone H4 (GL50803_135002 and 135003), but proteomic analyses showed that histones are actually down-regulated at the protein level in cysts [16]. This can be a preparation for the excystation process, which would suggest that the histone mRNAs are stored in the cysts, most likely in association with RNA binding proteins.

In line with earlier studies the *VSP* genes show large differences in gene expression during the late phase of encystation and most of all in cysts (Table S1). Most differential *VSP* genes are up-regulated in the cyst stage (130 *VSP* genes), and only a few are down-regulated (four *VSP* genes, Table S1). All the *VSP* genes that are up-regulated in cysts are down-regulated in trophozoites after excystation, except GL50803_14331 and GL50803_50363-VSP. There are only three DEGs: the two up-regulated VSPs and one down-regulated VSP (GL50803_137710-pVSP) that differ between the starting trophozoite population (T1) and the recently excysted trophozoite population (T2), showing an efficient resetting of gene expression back to the trophozoite expression profile from the cyst expression profile. This shows a connection between differentiation, both encystation and excystation, and antigenic variation in *Giardia*.

## 4. Discussion

A key factor for the transmission and survival of *Giardia* is cyst formation in order to continue the infection of new possible hosts. Earlier, there have been several efforts to characterize and understand the biological mechanisms involved in regulation of *Giardia* differentiation, especially encystation. Previous studies *in vitro* and *in vivo* have analyzed transcriptomics and proteomics with different methods shedding some light on lipid, sugar, and proteolytic metabolism during encystation [14,16,17,18]. However, past studies to characterize RNA expression during *Giardia* encystation were limited due to more basic technologies [12,15,19]. The study of few timepoints of this process furthermore limited the resolution and understanding of the bigger and more detailed picture of the whole process. This study aimed to expand and complement the current knowledge of what is occurring during encystation in *G. intestinalis*. A high-resolution gene expression map was built using deep RNA sequencing data of twelve timepoints during the whole-cell differentiation process and using the newly annotated genome of *G. intestinalis* WB [22] in the mapping of the reads to strengthen it even more. Our results divided the encystation process into early, mid, and late encystation. The general picture showed that the process occurs gradually with large changes in gene expression towards the late timepoints (Figure 1), as previously observed [15]. Non-clustered heat maps of known gene expression regulators show (Figure 2) that there is a gradual change in expression of transcription factors (Figure 2A) and chromatin regulators (Figure 2B). Our data confirm the important role of the Myb2 transcription factor early in encystation, and the number of genes regulated by Myb2 seems to be much larger than earlier described (Figure 3). Previously, the transcription factor Myb2 has been linked to the expression of important genes, such as *G6PI* and *CWP-1* and *-2*, during the early stages of encystation [13,15,34]. Earlier studies of RNA expression during encystation identified the presence of Myb2 binding sites up-stream of a small consensus set (13 genes) of early induced genes [13,15]. Our in-silico analysis of genes up-regulated during encystation suggest that the Myb2-regulon is much larger (Figure 3) and that this transcription factor is key for up-regulation of encystation-specific genes during at least three of the early-mid timepoints (7.5, 10.5, and 14 h). The conservation of the localization of the Myb2 binding motifs up-stream of the corresponding genes in other *Giardia* assemblages confirm its importance. The Myb2 transcription factor is regulated by phosphorylation by the cdk2 kinase [38], and here, we identified a putative cyclin (GL50803_15532) that is up-regulated very early but also late in the encystation process. Further detailed studies of the regulation of this cyclin-like protein can generate important information on the earliest stages of encystation. Several other transcription factors that earlier have been suggested to be involved in the regulation of the encystation process are induced in the mid (E2F/DP and ARID2) and late (GARP-like protein 2 and 3) stages, suggesting a transcription factor cascade type of regulation during cell differentiation similar to what can be seen in other eukaryotes, including other protozoan parasites [57,58,59]. Transcription factors, like Myb1 and GARP-like protein 4, are constitutively expressed on the RNA level from trophozoites to cysts (Figure 2A), but they still can be regulators of the encystation-specific genes, especially in the trophozoite stage; here, specific characterization is needed.

Contrary to what is observed with the transcription factors, especially with Myb2, we observed that changes in gene expression for chromatin modifiers and histones (Figure 2B) start from mid to late encystation showing the largest expression changes in the cyst stage, explaining the large global changes in gene expression profiles in the late stages of differentiation. However, we cannot rule out the role of epigenetic regulation of gene expression also early in encystation, especially in the silencing of the early induced genes, like the CWPs and Myb2 in trophozoites.

Earlier studies have shown an up-regulation of meiotic-related genes during the encystation process in *Giardia* [44,45]. Until now, no sexual stages have been identified in Giardia, and instead, homologous DNA recombination, supported by the meiotic-related genes, has been suggested to occur in an event that has been named diplomixis [45,46]. In diplomixis, the two *Giardia* nuclei fuse, and DNA is suggested to recombine between homologous chromosomes using at least three meiotic-related genes (*Spo11*, *Hop1*, and *Rad51*). When it comes to our analysis (Figure 4), we can see that the activation of meiotic-related genes starts at 10.5 h, and it continues gradually until the end of encystation, showing the most robust expression and abundant up-regulation of meiotic-related genes during the cyst stage. We can also detect an increased level of double-stranded DNA breaks around 10.5 h of *in-vitro* encystation. Our data suggest that the meiotic-related genes are expressed in a cascade-like fashion, and our results set the stage for further analyses of specific proteins in order to characterize the diplomixis process. 

Several gene families with up to 300 members of different cysteine-rich surface proteins (HCMPs, VSPs, and pVSPs) have been identified in the *Giardia* genome [22]. In our analysis, we looked at the expression of these different genes to analyze their periodicity and level of expression during encystation. The VSPs have been shown to be involved in antigenic variation as a mechanisms of protection for the parasite to avoid its elimination by the adaptative immune system of the host [60]. The VSPs have signal peptides, high cysteine content, conserved C terminal ends with one membrane-spanning helix, and a short 5 amino acid (CRGKA) cytoplasmic region, whereas the pVSPs lack the N terminal signal peptide [22]. Here, we identified the most up-regulated VSPs and pVSPs going from trophozoites to cysts (Figure 6), observing a higher level of expression during the cyst stage, which could be interpreted as a preparation of the parasite for excystation to be able to produce cells with new antigenic surfaces compared to the original population. HCMPs are a family of proteins still poorly characterized but with a resemblance to VSPs, except for comparatively larger cytoplasmic domains. One HCMP has been previously been shown to be up-regulated during encystation [61], and several are up-regulated during interactions with host cells *in vitro* [54]. In our study, we could identify several different expression profiles of HCMPs, with a few being up-regulated early and several being up-regulated late in encystation, and these changes are reset in excystation (Figure S2). Thus, it is clear that both encystation and excystation are connected to antigenic variation, both in the HCMP and VSP families. 

## Figures and Tables

**Table 1:** Number of DEGs at each timepoint during encystation and excystation of *Giardia*.

Time	Up	Down
3.5	6	14
7	21	12
10.5	78	9
14	200	23
17.5	382	130
21	598	414
24.5	700	575
28	905	770
31.5	1015	934
Cyst	1786	1623
T2	2	1

**Table 2:** GO term analyses of DEGs during encystation and excystation of *Giardia*.

Cluster	E-Score	No. Genes
**Up-regulated**		
**10.5–14 h**		
Fatty acid metabolism	1.9	3
**17.5–21 h**		
Fatty acid metabolism	1.3	3
**24.5–28 h**		
Lipid metabolic process	1.7	7
ATP binding	1.5	90
**31.5–Cyst**		
EGF-like	12.4	102
Amino acid transporter	1.4	9
**Down-regulated**		
**17.5–21 h**		
Nucleotide binding	5.4	35
Protein biosynthesis	2.9	8
Regulation of gene expression	1.9	3
ATP binding	1.5	73
**24.5–28 h**		
Nucleotide binding	4.5	49
Protein folding	4.4	10
Protein phosphorylation	1.8	24
**31.5–Cyst**		
Translation	4.23	41
ATP binding	3.6	237
Protein biosynthesis	1.8	23

## Data Availability

Raw files can be found at GEO [24] with accession number GSE141795 (https://www.ncbi.nlm.nih.gov/geo/query/acc.cgi?acc=GSE141795).

## Supplementary Materials

The following are available online at https://www.mdpi.com/article/10.3390/genes12121932/s1, Description of Supplementary files. Figure S1: Cyclin expression; Figure S2: HCMP expression; Figure S3: expression of enzymes involved in lipid metabolism; Figure S4: Analyses of double-stranded DNA breaks; Figure S5: Ribosome protein expression; Figure S6: Top 10 expressed VSP genes; Table S1: Differentially expressed genes during encystation; Table S2: Genes enriched in GO term analyses; Table S3: Analyses of Myb1 binding sites.
