# Supplementary material for: A Detailed Gene Expression Map of Giardia Encystation
Source: Genes (Basel). 2021 Nov 30;12(12):1932. doi: 10.3390/genes12121932 (PMC8700996; doi:10.3390/genes12121932)
Supplement: Supplementary file 1 [file genes-12-01932-s001.zip › Information supplementary files Rev 1.pdf]

Figure S1. Heat-map showing expression changes through-out encystation of cyclins. Expression changes are in log2 space and capped at -5 and 5 respectively to improve visualization. Upregulation is shown in red, while downregulation is shown in blue. For each gene the unique Geneid (starting in GL50803_) and the annotated function is given.

Figure S2. Heat-map showing expression changes through-out encystation of high cystein membrane proteins identified in previous work from our lab (Peirasmaki *et al., 2020)*. Expression changes are in log2 space and capped at -5 and 5 respectively to improve visualization. Upregulation is shown in red, while downregulation is shown in blue. For each gene the unique Geneid (starting in GL50803_) and the annotated function is given.

Figure S3. Heat-map showing expression changes through-out encystation of very long chain fatty acid biosynthesis genes. Expression changes are in log2 space and capped at -5 and 5 respectively to improve visualization. Upregulation is shown in red, while downregulation is shown in blue. For each gene the unique Geneid (starting in GL50803_) and the annotated function is given.

Figure S4. Analysis of the presence of DNA double breaks using the antibody H2AX and Aphidicolin as control, **S4A)** Graphs showing the percentage of cells with double breaks during diferent time points throughout encystation first in a combination with a CWP2 antibody and second only with the H2AX antibody, thirdly a Western Blot showing the detection of the H2AX in cell extracts from different time points during encystation (Aphidicolin, Trophozoites, 3.5h, 7h, 10.5h, 14h, 17.5h, 21h and 24h). **S4B)** Examples of the identification DNA double breaks during cell differentiation at different time points (Aphidicolin, Trophozoites, 3.5 h, 14h, 17.5 h 24.5 h) by immunofluorescence using the H2AX antibody (red). Scale bars represent 20 μm.

Figure S5. Heat-map showing expression changes through-out encystation of elongation factors, translation initiation factors and ribosomal proteins. Expression changes are in log2 space and capped at -5 and 5 respectively to improve visualization. Upregulation is shown in red, while downregulation is shown in blue. For each gene the unique Geneid (starting in GL50803_) and the annotated function is given.

Figure S6. Heat-map showing expression changes through-out encystation of the top 10 most highly induced VSPs and pVSPs. Expression changes are in log2 space and capped at -5 and 5 respectively to improve visualization. Upregulation is shown in red, while downregulation is shown in blue. For each gene the unique Geneid (starting in GL50803_) and the annotated function is given.
