# Supplementary figures and images for: A Detailed Gene Expression Map of Giardia Encystation
Source: Genes (Basel). 2021 Nov 30;12(12):1932. doi: 10.3390/genes12121932 (PMC8700996; doi:10.3390/genes12121932)

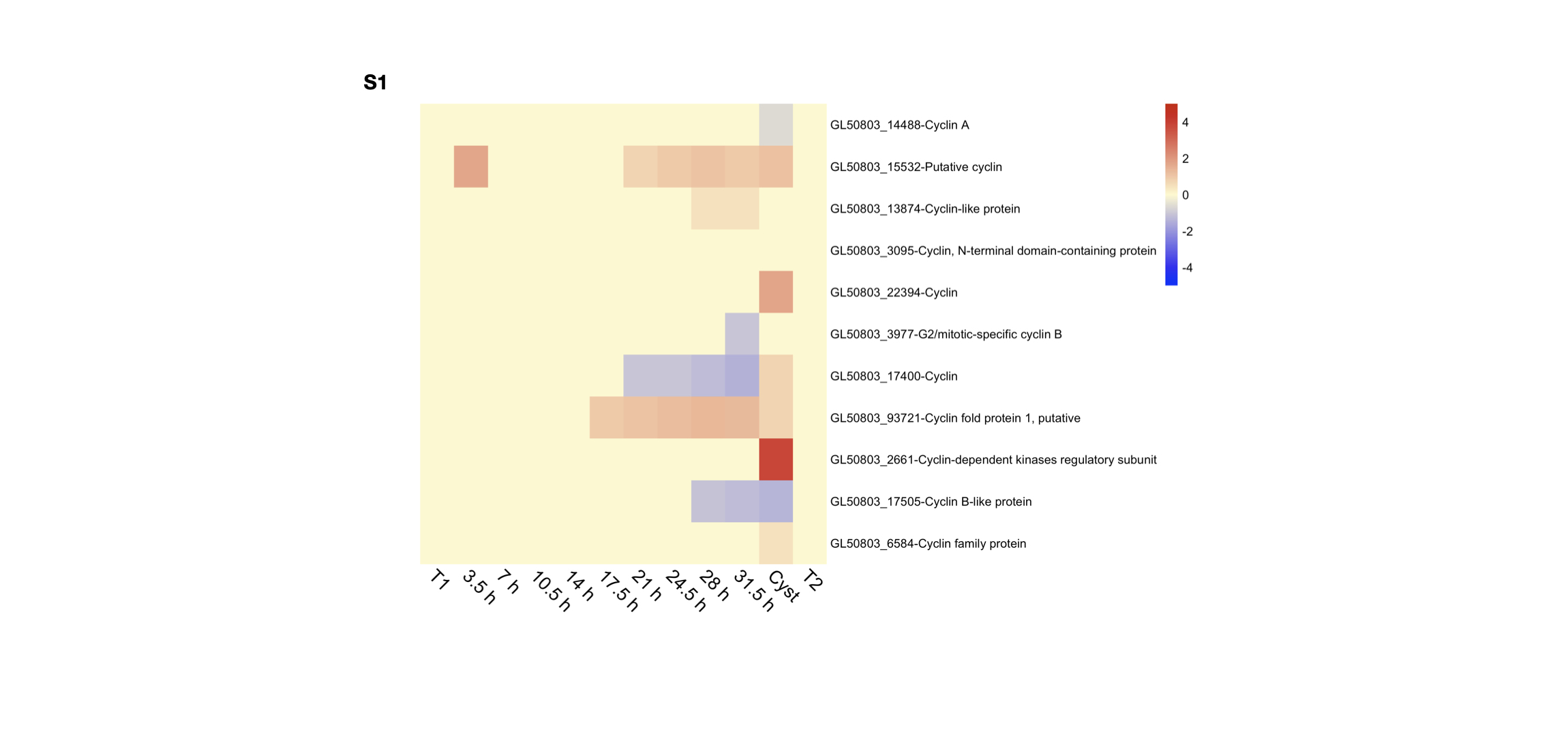

Supplement: Supplementary file 1 [file genes-12-01932-s001.zip › Supplementary figure S1.jpeg]

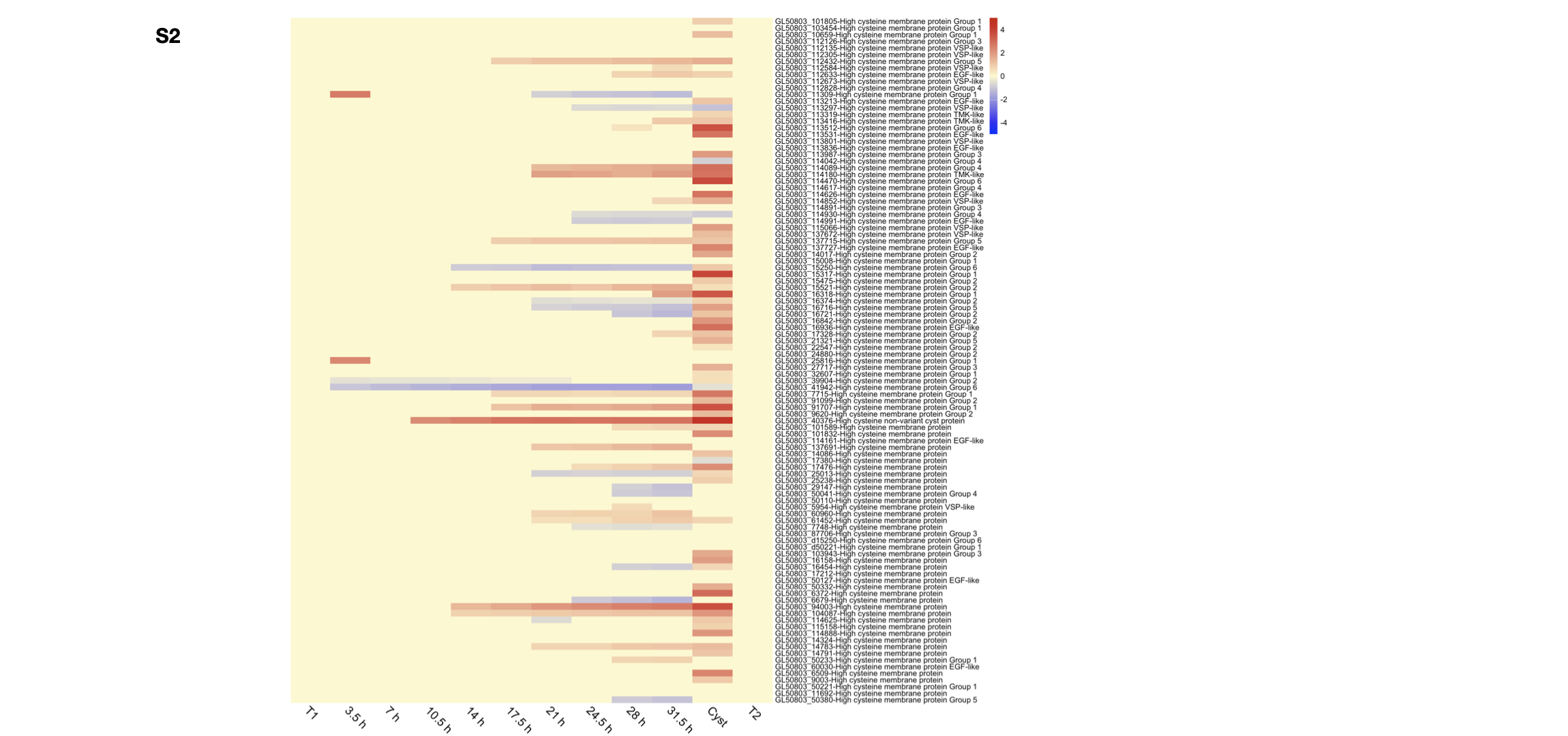

Supplement: Supplementary file 1 [file genes-12-01932-s001.zip › Supplementary figure S2.jpeg]

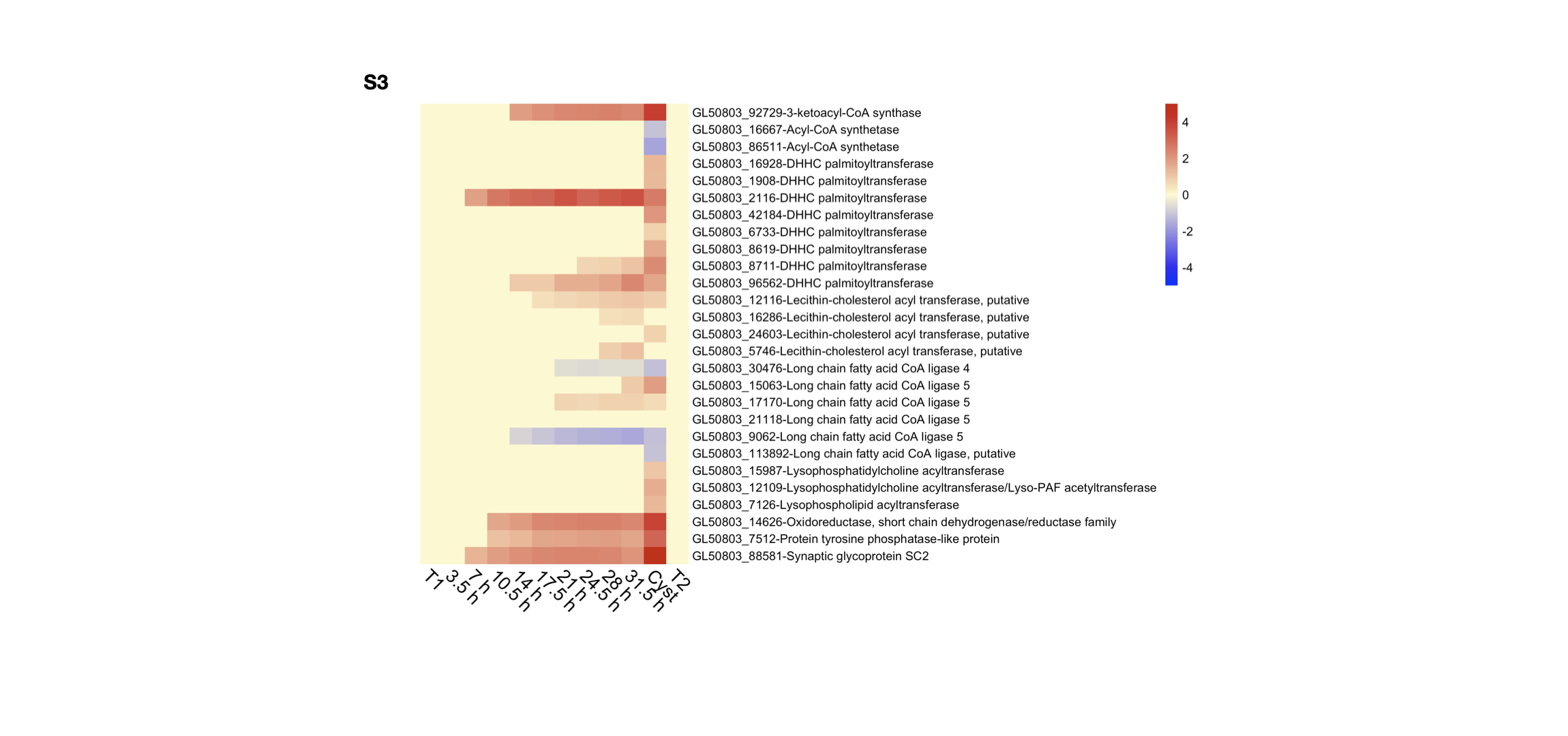

Supplement: Supplementary file 1 [file genes-12-01932-s001.zip › Supplementary figure S3.jpeg]

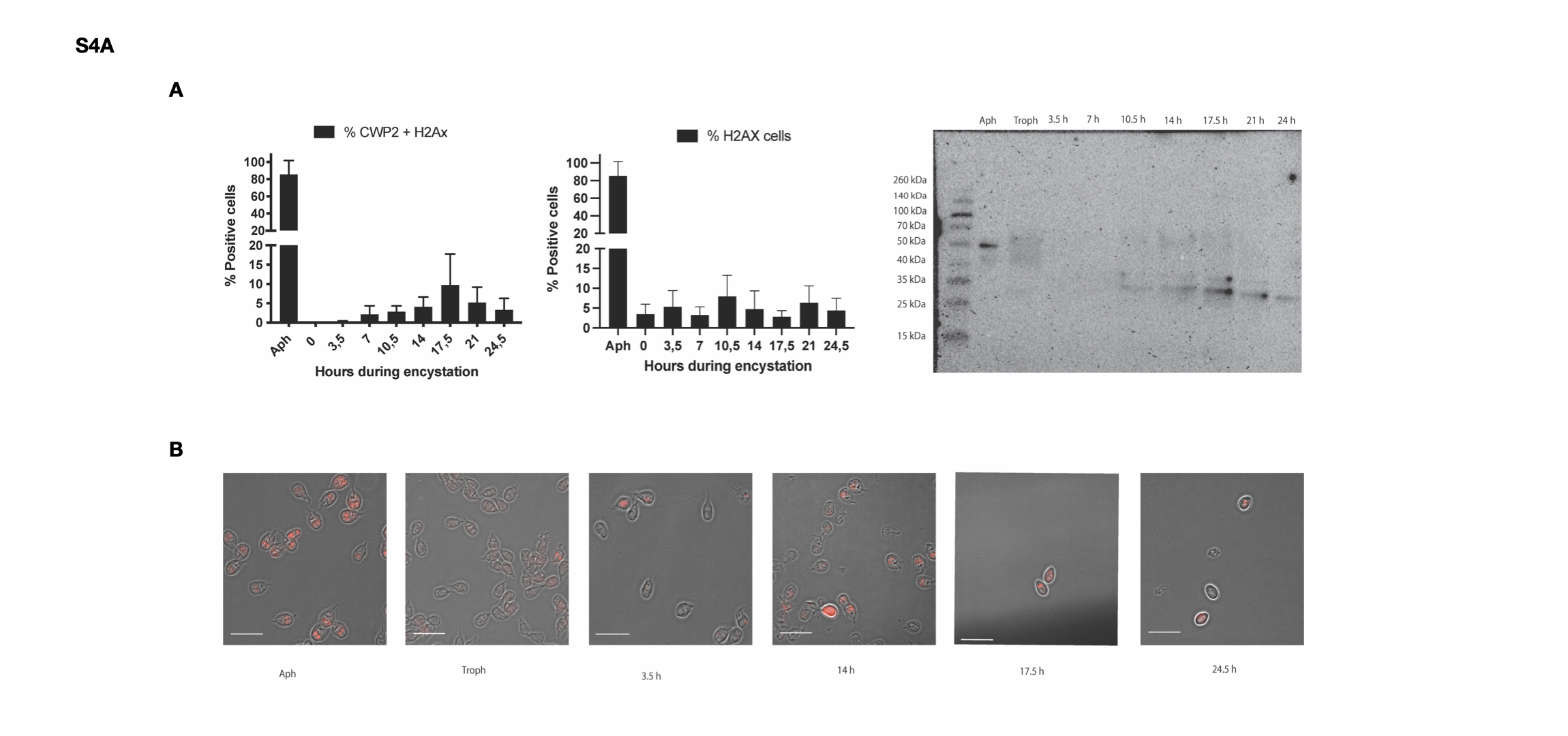

Supplement: Supplementary file 1 [file genes-12-01932-s001.zip › Supplementary figure S4.jpeg]

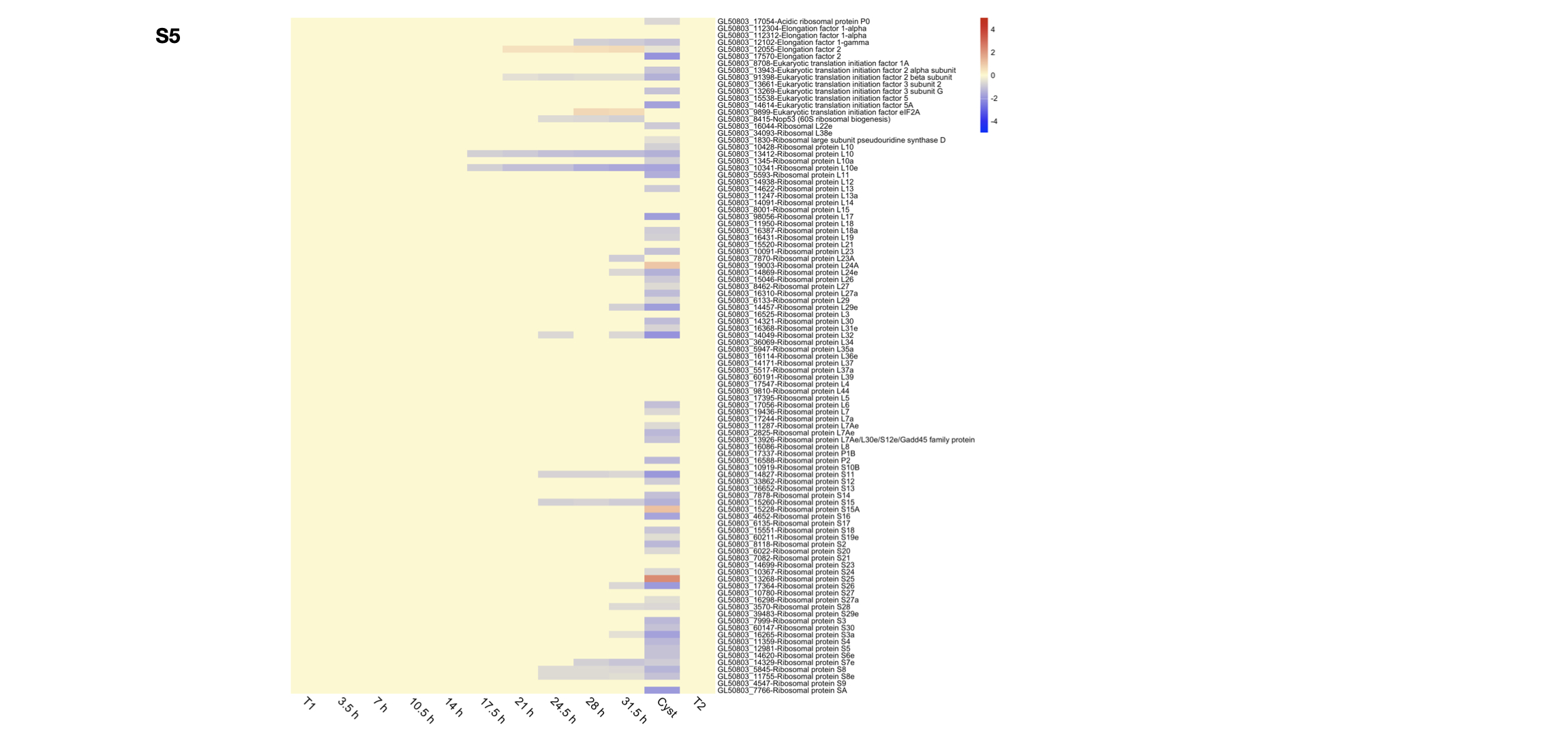

Supplement: Supplementary file 1 [file genes-12-01932-s001.zip › Supplementary figure S5.jpeg]

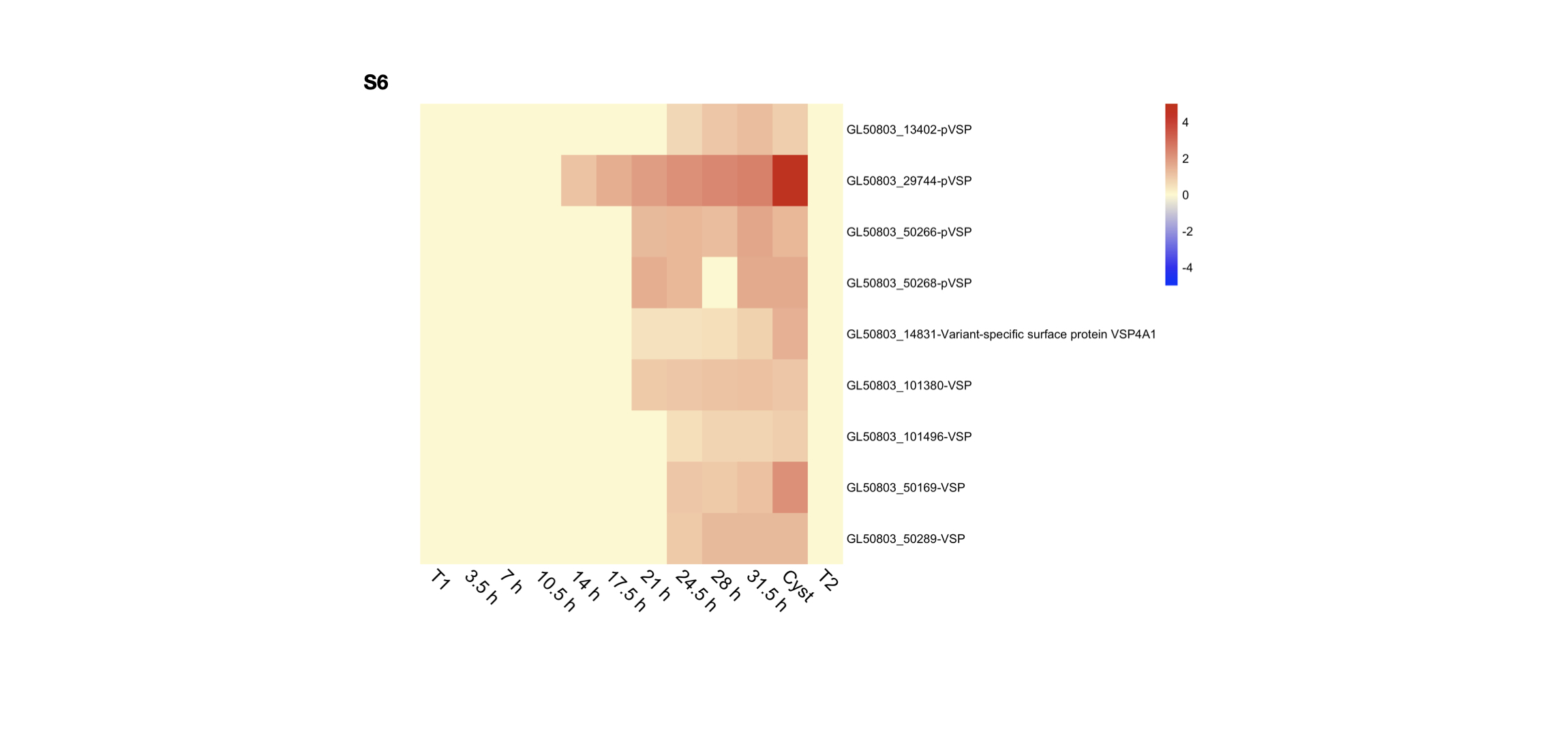

Supplement: Supplementary file 1 [file genes-12-01932-s001.zip › Supplementary figure S6.jpeg]
